# Summer and winter habitat suitability of Marco Polo argali in southeastern Tajikistan: A modeling approach

**DOI:** 10.1016/j.heliyon.2017.e00445

**Published:** 2017-11-06

**Authors:** Eric Ariel L. Salas, Raul Valdez, Stefan Michel

**Affiliations:** a Department of Fish, Wildlife and Conservation Ecology, New Mexico State University, Las Cruces, NM 88003, USA; b IUCN Species Survival Commission, Caprinae Specialist Group, Kannawurf, Germany

**Keywords:** Ecology, Evolution, Zoology, Environmental science, Geography, Biological sciences

## Abstract

We modeled summer and winter habitat suitability of Marco Polo argali in the Pamir Mountains in southeastern Tajikistan using these statistical algorithms: Generalized Linear Model, Random Forest, Boosted Regression Tree, Maxent, and Multivariate Adaptive Regression Splines. Using sheep occurrence data collected from 2009 to 2015 and a set of selected habitat predictors, we produced summer and winter habitat suitability maps and determined the important habitat suitability predictors for both seasons. Our results demonstrated that argali selected proximity to riparian areas and greenness as the two most relevant variables for summer, and the degree of slope (gentler slopes between 0° to 20°) and Landsat temperature band for winter. The terrain roughness was also among the most important variables in summer and winter models. Aspect was only significant for winter habitat, with argali preferring south-facing mountain slopes. We evaluated various measures of model performance such as the Area Under the Curve (AUC) and the True Skill Statistic (TSS). Comparing the five algorithms, the AUC scored highest for Boosted Regression Tree in summer (AUC = 0.94) and winter model runs (AUC = 0.94). In contrast, Random Forest underperformed in both model runs.

## 1 Introduction

One effective approach to guide conservation efforts for terrestrial species is the establishment of habitat suitability models. These species distribution models (SDMs) are normally used for predicting suitable habitats (Gionfriddo and Krausman, 1986; Smith et al., 1991; Andrew et al., 1999; Bangs et al., 2005), animal abundance (Bristow and Crabb, 2008), separation of habitats between species (Kissell et al., 1996), restoration of large mammals (Johnson, 1995), and habitat connectivity (Gagnon et al., 2013). They are valuable spatial ecological tools to better assess the relationship between species distributions and environmental factors, and understand future steps for species management and policy (Elith and Leathwick, 2008; Salas et al., 2017). Previous studies in the Tibetan plateau have applied SDMs specifically for mountain ungulates such as the wild yak (*Bos mutus*), chiru (*Pantholops hodgsonii*), kiang (*Equus kiang*), Tibetan gazelle (*Procapra picticaudata*), and Przewalski's gazelle (*Procapra przewalskii*) (Schaller, 1998; Luo et al., 2015) and found differences in species richness between the southern and northern mountain regions of the plateau. In North American ungulates, Cunningham (1989) and Zeigenfuss et al. (2000) applied SDMs to assess habitat suitability for bighorn sheep in the Mojave Desert, Arizona and Colorado Plateau Desert, respectively. A model that used expert-opinion by Rubin et al. (2010) in the peninsular ranges of southern California, showed vulnerability of bighorn sheep to lack of habitat connectivity. While these models performed well in areas for which they were intended, they may be poor predictors when applied to predict habitat suitability in other areas (Cunningham, 1989; Wenger and Olden, 2012). Probable reasons for this are the selection of model predictors (Zeigenfuss et al., 2000), inaccurate processing of variables (e.g., Normalized Difference Vegetation Index, NDVI) from remotely-sensed data (Borowik et al., 2013; Wen et al., 2016), and incomplete coverage of species-environment response curves by the presence data used in the model.

Argalis (*Ovis ammon*) are wild sheep restricted to Asia in Afghanistan, China, Kazakhstan, Kyrgyzstan, Mongolia, Pakistan, Russia, Tajikistan and Uzbekistan (Valdez and Weinberg, 2011). Marco Polo argali (*O. a. polii*) occur in eastern Tajikistan and adjacent areas of surrounding countries: China, Afghanistan, Kyrgyzstan, Pakistan. They are highly desired big game trophies because of their long horns of up to 191 cm (75 in). Argali principally occupy undulating terrain lacking tall vegetation but with a rugged surface and use the precipitous component for escape. Escaping argali normally go up slopes and move out of sight of the perceived danger. Argalis are listed as endangered by the U.S. Fish and Wildlife Service throughout their range, except in Kyrgyzstan, Tajikistan, and Mongolia where they are designated as threatened. They are listed in CITES Appendix II and as Near Threatened in the IUCN Red List. Argalis have declined in numbers and distribution during the last century (Harris and Reading, 2008; Valdez and Weinberg, 2011). However, because of their economic value as hunting trophies, hunting concessions and community-based conservancies prohibit illegal hunting and as a consequence, argali in Tajikistan have significantly increased in recent years. Tajikistan has greater numbers of argali than any other country with a minimum of 24,000 in the Pamirs (Michel and Muratov, 2010; Valdez et al., 2016).

In the case of the central Asian argali wild sheep, there were only three published argali habitat modeling studies (Singh et al., 2009; Chimeddorj et al., 2014; Khan et al., 2015). Khan et al. (2015) analyzed Marco Polo sheep habitat near the transboundary area between China and Pakistan using habitat suitability index (HSI) in Maxent (Phillips et al., 2006). The study was conducted in two locations: the Khunjerab National Park (KNP) in Pakistan’s Karakoram and the Taxkorgan Nature Reserve (TNR) in China’s Quorum and Pamir mountains. Based on a dataset of small sample size, Khan et al. (2015) showed that Marco Polo sheep distribution was significantly associated with low temperature during early winters and higher NDVI (vegetation). Chimeddorj et al. (2014) explicitly modeled suitable habitat for argali populations across the border regions of Mongolia and Russia by estimating resource selection function (RSF). Chimeddorj et al. (2014) only used three variables in the model − elevation, ruggedness index (RI), and distance to state border (all three variables were positively correlated) − and acknowledged that other parameters could have helped improve the understanding of habitat selection by argali. Singh et al. (2009) also used resource selection function (RSF) to model the habitat of the argali in the Indian Transhimalaya. Singh et al. (2009) used five habitat variables: elevation, ruggedness index, NDVI, aspect, and slope. Among the five, elevation was the most important variable determining the habitat suitability of argali. The variables used by Singh et al. (2009), Chimeddorj et al. (2014), and Khan et al. (2015) in modeling the argali habitat in mountainous central Asian regions, were utilized in this study in addition to other environmental predictors of habitat that we deemed important for the Marco Polo argali in Tajikistan.

Current conservation efforts in the Pamirs of Tajikistan are focused on (1) community-based conservation and management of mountain ungulates and on snow leopard (*Panthera uncia*), which exists in a healthy population (Michel and Rosen, 2016), and (2) the removal and reducing the risk of teresken (*Krascheninnikovia ceratoides*), a dwarf shrub that is extensively used as a fuel substitute, and the resulting loss of vegetative cover (Kraudzun, 2012; Zandler et al., 2015; Salas et al., 2016b). A major concern is the increase of forage competition for argali by growing domestic livestock numbers and resulting rangeland degradation. Apart from these efforts, there is a need to assess the status of argali in the region, especially, considering most of the available information is based on visual surveys (Harris and Reading, 2008; Michel and Muratov, 2010; Valdez and Weinberg, 2011; Valdez et al., 2016). Unfortunately, these surveys do not quantitatively identify the factors that influence or limit argali habitat suitability.

We applied and compared five statistical modeling algorithms and tested several predictors to predict the distribution of argali in our study area. We focused the modeling approach to argali populations during the summer and winter seasons, utilizing data collected from 2009 to 2015. Our study objectives were two-fold: (1) to determine which algorithm is more reliable in predicting wild sheep occurrence and whether the algorithm prevails for summer and winter habitats, and (2) to evaluate which variables are relevant for summer and winter models.

## 2 Materials and methods

**Fig. 1:**
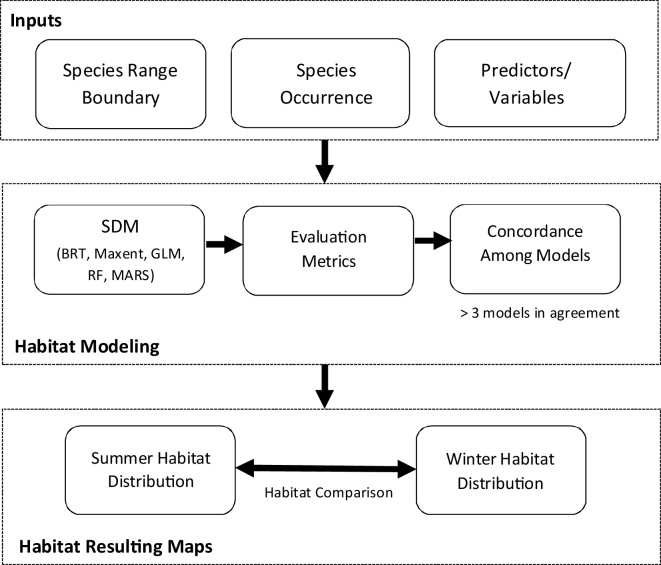
Schematic framework for generating and validating the summer and winter models of habitat suitability for argali populations in the southeastern Pamir region of Tajikistan.

The methodology was based upon the steps displayed in Fig. 1. The following provides a summary of data sources, the variables used in the models, model structure, and assessment algorithm.

### 2.1 Study area

**Fig. 2:**
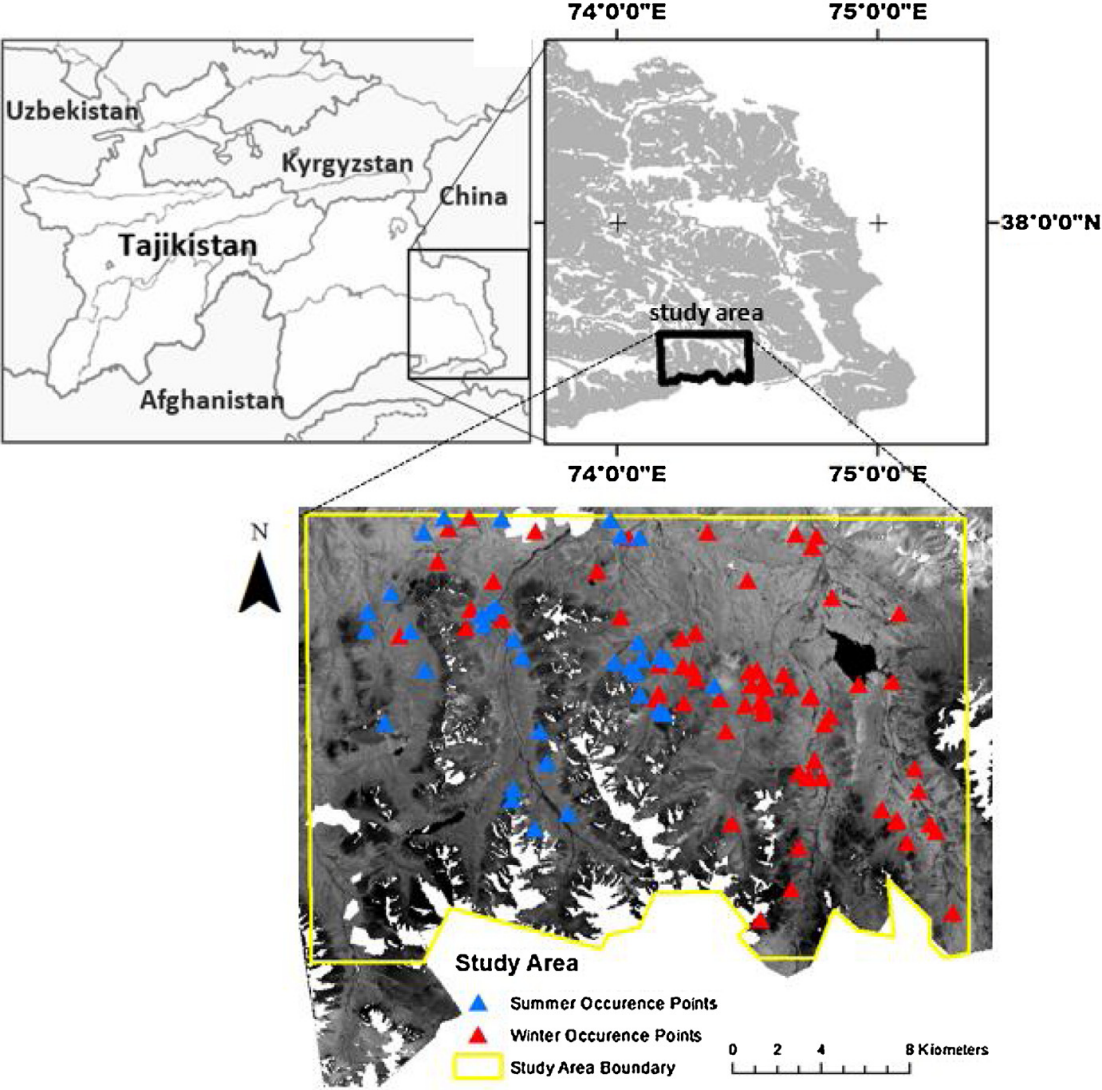
Location of study area in the southeastern Pamir region of Tajikistan (with argali occurrence points overlaid). The Central Asian country of Tajikistan is bordered by Afghanistan, China, Kyrgyzstan and Uzbekistan. Note: the base layer used in the zoomed-in map is the July 2014 Landsat image.

The study area (Fig. 2) is located in the Pamir Mountains of southeastern Tajikistan in the Gorno-Badakhshan Autonomous Region adjacent to the border with Afghanistan and the Little Pamir (Pamir-e Khurd), between latitudes 37°N to 38°N and longitudes 74°E to 75°E, and covers an area of approximately 2230 km^2^. The rocky mountainous terrain is at an elevation of 3500 m to 5500 m above mean sea level. Average annual precipitation is 100 mm with subzero average temperatures from October to March. The study area covers parts of a larger sport hunting concession primarily for Marco Polo sheep (Valdez et al., 2016), with a minimum population of 8,000 wild sheep. The only other wild ungulate species is the Siberian ibex (*Capra sibirica*). Wild predators include wolf (*Canis lupus*), red fox (*Vulpes vulpes*), brown bear (*Ursus arctos isabellinus*) and snow leopard. There is no mining activity, no large (> 50 people) villages, no paved roads, and no fences in the study area. The study site can become inaccessible in the winter because of heavy snow accumulation. The area is patrolled to minimize illegal hunting (Valdez et al., 2016).

Domestic ungulates include sheep (*Ovis aries*), yak (*Bos grunniens*), and goat (*Capra hircus*); domestic sheep are the most numerous followed by yak and few goats. Domestic animals, except for yak and few herds of sheep and goats, are moved to lower pastures during the fall, winter, and early spring (October–May) because of the harsh winter conditions at higher elevations. Except for small herds of yaks, domestic animals were not present in the study area during summer and winter surveys. The dominant plant species are semishrubs such as teresken and sagebrush (*Artemisia* spp.); grasses (e.g., *Poa* spp.*, Festuca* spp.*, Hordeum* spp.*, Elymus* spp.); sedges (*Carex* spp. and *Kobresia* spp.); and forbs (e.g., *Dracocephalum* spp., *Oxytropis* spp., *Astagalus* spp., *Acantholimon* spp., *Crepis flexuosa*, and *Potentilla* spp.) (Valdez et al., 2016).

### 2.2 Argali data

Argali occurrences were collected from winter and summer field surveys over multiple years. Sheep presence points were direct field observations from randomly surveyed potential habitat of the species, observed at distances 300 m to 1500 m, and carried out as part of previously conducted studies (Michel and Muratov, 2010; Valdez et al., 2016). Summer surveys were conducted in August 2010 and September 2013. Winter surveys were done in December 2009 and March 2015. During summer, mostly ewes were recorded. Adult males were fewer, probably because they occur at higher elevations and are segregated from ewes. During winter, both adult ewes and males were recorded; at this season, which is the mating period, males and females occur in common herds. Because clustered occurrences could introduce potential bias, we removed multiple presence localities in a 30 m x 30 m grid and analyzed a single occurrence per pixel (Talbert, 2012). A total of 35 and 64 occurrences were used for summer and winter models, respectively.

### 2.3 Landsat data

We downloaded three Landsat images with minimal cloud cover (path/row: 150/34), two for the summer season (July 2008 and 2014) and one for the winter season (January 2010) as part of the predictor variables. We used two images for summer to capture possible vegetation changes in argali locations. We downloaded the 30-m spatial resolution Landsat images from the U.S. Geological Survey Earth Resources Observation and Science (USGS EROS, https://eros.usgs.gov/usa) resource archive. Preprocessing of the images is necessary to enhance the quality of the data and to remove inherent noise that can have negative impacts on the classification. For the 2008 Landsat scene, we filled the Scan Line Corrector (SLC) data gaps. We normalized the images by converting the measured digital number (DN) values to top of atmosphere (TOA) reflectance to remove variations between images caused by sensor differences, Earth-sun distance, and solar zenith angle (Chander and Markham, 2003). Screening of cloud patches, cloud shadows, and mountaintop snow was performed on the summer images to ensure that the image was devoid of obstructions that may result in false classification. In the case of clouds, we conducted visual and/or spectral examinations of the image to assess for cloud presence and shadow contaminations, delineating them and masking from the analysis when present. For the winter image, we used the improved Fmask algorithm (Zhu et al., 2015) for Landsat 7 to isolate the snow cover in the study area. All image processing were done using the ENVI software (ITT, 2014).

### 2.4 Predictor variables

For the summer Landsat images, we calculated spectral indices. Apart from NDVI (Guyot and Gu, 1994) as index of forage abundance (Singh et al., 2009), we derived the Modified Soil-adjusted Vegetation Index (MSAVI) that could aid in feature discrimination (Palacio-Prieto and Luna-Gonzalez, 1994; Salas et al., 2015). Furthermore, we added digital elevation model (DEM), continuous slope, and aspect layers to depict the terrain components of the study area (Parolo et al., 2008). For escape terrain, we created a continuous distance around polygon patches with slopes ≥ 30° (Smith et al., 1991; Turner et al., 2004). Bleich et al. (2009) found that proximity to escape terrain could be the main predictor of habitat selection for mountain ungulates. The processed DEM with a 1 arc-second, or about 30 meters (98 feet) resolution was sourced from NASA’s Shuttle Radar Topography Mission (SRTM) digital elevation dataset that is available for download online (USGS 2016). Other datasets that could impact species visibility and considered as important in the modeling, such as continuous distances from riparian areas and vegetation distribution (Demarchi et al., 2000; DeCesare and Pletscher, 2006; Salas et al., 2015; Salas et al., 2016a), were also included as predictors. To further capture the features of the landscape terrain, the terrain ruggedness index (RI) was calculated from the DEM based on Sappington et al. (2007). The RI shows the average change in elevation between a center pixel and its eight neighboring pixels in a 3 by 3 window. Turner et al. (2004) showed that terrain ruggedness could be a better predictor than proximity to escape terrain when both are used in the same modeling set. In other ungulate habitat evaluation studies (Taylor et al., 1998; Rubin et al., 2010), topographic variables consistently predicted habitat selection. Further, we added Landsat bands to further separate classes, e.g., vegetation surfaces from soil and rock (Salas et al., 2016b). Table 1 lists selected datasets and their derivatives, as well as the vegetation index equations used as predictors in the habitat suitability modeling. All GIS-based analyses, like deriving the aspect variable from DEM, were performed using the ArcGIS 10.3 software (ESRI, 2013).

**Table 1:** Input variables and derivatives used in the suitability habitat modeling of argali wild sheep in the Tajikistan Pamirs. An asterisk (*) denotes final variables used for modeling the habitat. NDVI = Normalized Difference Vegetation Index; MSAVI = Modified Soil-Adjusted Vegetation Index; DEM = Digital Elevation Model; RI = Roughness Index.

Band	Wavelength (μm)	Application
Landsat 7*		
Band 1	0.45 − 0.52	Differentiates soil/rocks from vegetation
Band 2	0.52 − 0.60	More separation of vegetation from soil
Band 3	0.63 − 0.69	Provides strong chlorophyll absorption region and strong reflectance region for most soils
Band 4	0.77 − 0.90	Crop identification and health, delineate water
Band 5	1.55 − 1.75	Detection of snow, clouds, stresses vegetation
Band 7	2.09 − 2.35	Region for soil and rock, water absorption region
Band 6	10.40 − 12.50	Thermal region
**Landsat 8**		
Band 1	0.43 − 0.45	Coastal aerosol
Band 2	0.45 − 0.51	Differentiates soil/rocks from vegetation
Band 3	0.53 − 0.59	More separation of vegetation from soil
Band 4	0.64 − 0.67	Provides strong chlorophyll absorption region and strong reflectance region for most soils
Band 5	0.85 − 0.88	Crop identification and health, delineate water
Band 9	1.36 − 1.38	Detection of clouds
Band 6	1.57 − 1.65	Detection of snow, clouds, stresses vegetation
Band 7	2.11 − 2.29	Region for soil and rock, water absorption
Band 10	10.60 − 11.19	Thermal region
Band 11	11.50 − 12.51	Thermal region
**Index**	**Equation**	**Application**
NDVI*	$\frac{\left(NIR-Red\right)}{\left(NIR+Red\right)}$	Measure of greenness
MSAVI*	$\frac{\left(NIR-Red\right)\left(1+0.5\right)}{NIR+Red+0.5}$	Vegetation, but more for soil background
**Topo Feature**	**Equation**	**Application**
DEM*		Elevation data
RI*	$\sqrt{Abs\left({\left(\%Max\right)}^{2}-{\left(\%Min\right)}^{2}\right)}$	Change in elevation
Slope*		Percent slope
Aspect*		Direction the slope faces
Vegetation distribution*		Spatial distribution of green summer vegetation
Distance from riparian areas*		Provides continuous distance from identified riparian areas
Distance to escape terrain*		Provides continuous distance from a defined slope of ≥ 30°
**Others**		
Snow cover*		Defines the extent of the snow cover

### 2.5 Habitat suitability modeling

We tapped the USGS modeling tool Software for Assisted Habitat Modeling (SAHM) for VisTrails (Talbert, 2012) to predict probability of argali occurrence, based on our presence data. Rather than utilizing one species distribution model (SDM) (Phillips and Dudík, 2008; Chimeddorj et al., 2014), we fitted the following SDMs: Generalized Linear Model (GLM); Random Forest (RF) (Breiman, 2001; Liaw and Wiener, 2002); Boosted Regression Tree (BRT) (Elith et al., 2008); Maxent (Phillips et al., 2006); and Multivariate Adaptive Regression Splines (MARS) (Leathwick et al., 2006) for comparisons based on their performance with presence-only data (Elith et al., 2006). The GLM is a linear regression adapted to binary count data. The method uses stepwise procedure to select covariates in the model. The MARS non-parametric algorithm build flexible models by fitting piecewise logistic regressions. Though it has similarities with GLM, MARS is better in accommodating nonlinear responses to predictors and at the same time lessens the effects of outlying observations. The model RF uses decision trees through random grouping of the covariates. Random forest models both interactions of the variables and their nonlinear relationships and does not split the data into training and test as RF utilizes bootstrapping to fit individual trees (Breiman, 2001). Like the Random Forest, BRT also uses decision trees, but the method is robust to missing observations. BRT uses cross-validation by choosing models based on model comparisons of evaluation metrics (Elith et al., 2008). Maxent, a machine learning algorithm, minimizes relative entropy between the probability densities for the species and the one estimated for the available environment (Elith et al., 2011). It works best for presence-only modeling. While observed absence is valuable in modeling, data is oftentimes not available and using only presence data is unavoidable (Talbert, 2012). For species lacking absence data, SAHM tool randomly generated 10,000 background points (i.e., pseudo-absences) (Phillips and Dudík, 2008; Talbert, 2012). The tool takes the input field data and creates a binary mask for generation of background points using a Kernel Density Estimate (KDE) of the presence points with options for optimizing bandwidth. Background surfaces permit sampling of pixels where presence have been recorded in more of a used available specification instead of presence/pseudo-absence specification (Talbert, 2012). Our method does not guarantee the full removal of biases associated to sampling effort. Currently, there is no method that could. We combined individual models to reduce problems inherent in each algorithm. The combination entailed summing five binary maps generated from each statistical modeling algorithm (Lobo et al., 2008; Stohlgren et al., 2010). We used specificity = sensitivity (presence and absence have an equal chance of being correctly predicted) as the threshold in discretizing the probability maps, which was previously been identified as the optimal threshold (Liu et al., 2005). Sensitivity refers to the proportion of actual presences correctly classified as present by the model, while specificity refers to the proportion of actual absences correctly classified as absent. The final combinations consisted of pixel values that showed the number of models in agreement that a particular pixel is suitable for the species.

Concordance among the different distribution models is an assessment of confidence in model results. For instance, a combination score of zero means that no model has predicted the area to be suitable for the argali, while a score of five means that all five models agreed that the location is a suitable habitat. Rehfeldt et al. (2012) determined that environmental conditions were suitable for a species when three or more (at least 60%) of algorithms were in agreement. To further evaluate the performance of the modeling algorithms, we evaluated various measures of model performance, including the Area Under the Receiver Operating Characteristic (ROC) Curve (AUC) for the test data and correct classification rate (Fielding and Bell, 1997) and the True Skill Statistic (TSS) (Allouche et al., 2006). Swets (1988) classified values of AUC: those >0.9 indicated high accuracy, from 0.7 to 0.9 indicated good accuracy, and those <0.7 indicated low accuracy. The AUC value is the probability that the model would rank a randomly chosen presence observation higher than the randomly chosen absence observation. The TSS is presented as an improved measure of model accuracy that, unlike the commonly-used kappa statistics (Cohen, 1960), is not dependent on species prevalence (i.e. proportion of occurrence points for which the species is present) (Allouche et al., 2006). It should be noted that when comparing these measures for different models, several considerations need to be taken into account. For instance, AUC is reliant on the extent of the study area, the process of how the occurrence points were collected, prevalence, grain size, and whether the species being modeled is a generalist or specialist. Lobo et al. (2008) recommended that these parameters must be fixed in order for comparison among modeling algorithms. In addition, the likelihood of these evaluation metrics to be overestimated is high when the evaluation data is dependent on calibration data. In running the models, we used a 70–30 ratio. That means that 70% of the sample points were used to train the model, and 30% of the points were held out to test the model's performance. Also, our algorithm stratify the split by the response. That is, the ratio of presence to absence points should be nearly equal in the testing and training split (Talbert, 2012).

Because high multicollinearity among predictors was likely to manifest, the SAHM package was used to determine the most important variables based on the species occurrence. Highly correlated variables (r > 0.7) (Dormann et al., 2013) were removed because they can impede the interpretation of species-environment relationship. Our algorithm produces calibration and evaluation plots. The calibration plot shows the predicted probability of occurrence plotted against the actual proportions of occurrence. A logistic regression model is fit to the logits of the predicted probabilities of occurrence and is shown on the plot. This plot is used to determine how reliably a model will predict if a site is occupied or unoccupied. The evaluation plot shows the relationship between sensitivity and specificity as the threshold for discretizing continuous predictions into presence absence is varied. It also shows several standard plots for assessment of model residuals. Finally, we checked the behavior of the resulting cross-calibration plots to determine if the models tended to over or underpredict habitat suitability. We checked other qualitative assessments of model performance including deviance of residual plots. Deviance of residual plots are used to identify individual data points that may require further inspection or whether there may be an important environmental layer missing from the model inputs.

## 3 Results

### 3.1 Model performance

The performances of the five statistical models are shown in Table 2. Among all models, the AUC scores were highest for the BRT for both the summer (AUC = 0.94) and winter modeling runs (AUC = 0.94) followed by Maxent (AUCs 0.93 and 0.90 for summer and winter models, respectively), then MARS (AUCs 0.88 and 0.84), GLM (AUCs 0.81 and 0.82), and the RF (AUCs 0.74 and 0.76). Except for RF, the AUC for all models showed good predictive power with AUC ≥ 0.80. The values of percent correctly classified (%Correct) were also the high for BRT (88.4 and 88.7, respectively for the summer and winter data) and Maxent (86.5 and 84.3, respectively for the summer and winter data). For the True Skill Statistic, only BRT and Maxent had values above 0.70, while RF scored the lowest (TSS = 0.53 and 0.55 for the summer and winter modeling).

**Table 2:** The Area Under the Curve (AUC) associated with the test data, the percentages of occurrence points correctly classified (%Correct), and True Skill Statistics for the five models and for the summer and winter seasons in the Tajikistan Pamirs. Model abbreviations are as follows: GLM = Generalized Linear Model, MARS = Multivariate Adaptive Regression Splines, BRT = Boosted Regression Tree, and RF = Random Forest.

Measure	GLM	MARS	BRT	RF	Maxent
**Summer**					
AUC	0.81	0.88	0.94	0.74	0.93
%Correct	78.4	79.3	88.4	71.2	86.5
TSS	0.58	0.67	0.79	0.53	0.73
**Winter**					
AUC	0.82	0.84	0.94	0.76	0.90
%Correct	77.4	79.3	88.7	72.9	84.3
TSS	0.64	0.68	0.83	0.55	0.73

### 3.2 Habitat selection and variable importance

In modeling summer habitat, the distance to riparian areas was the most important predictor for four of the five SDMs (Table 3). NDVI was equally important, with GLM ranking the variable first and BRT, MARS, and Maxent placed the variable second. Landsat band 6 (surface temperature) and RI both ranked in the top five. The vegetation layer was the least important among the top five variables for summer habitat, for four of the five SDMs. The winter habitat modeling showed two different important variables at the top compared to the summer habitat (Table 3). Except for GLM, during winter season, slope was the best predictor of argali habitat. GLM picked MSAVI as the most important variable and slope was third. Aspect ranked fourth in four of the five SDMs.

**Table 3:** The top five important suitable habitat predictors for each statistical algorithm for summer and winter models of wild sheep habitat use in the Tajikistan Pamirs. The two most important variables for summer are NDVI and distance to riparian areas. For winter, the two most important variables are slope and Landsat band 6 (surface temperature). BRT = Boosted Regression Tree, GLM = Generalized Linear Model, MARS = Multivariate Adaptive Regression Splines, Maxent, and RF = Random Forest.

Rank	BRT	GLM	MARS	Maxent	RF
**Summer**					
1	Riparian	NDVI	Riparian	Riparian	Riparian
2	NDVI	RI	NDVI	NDVI	Aspect
3	Band 6	Riparian	RI	Band 6	NDVI
4	RI	Band 6	Band 6	RI	Band 6
5	Vegetation	Vegetation	Vegetation	Aspect	Vegetation
**Winter**					
1	Slope	MSAVI	Slope	Slope	Slope
2	RI	Band 6	Band 6	Band 6	Aspect
3	MSAVI	Slope	RI	MSAVI	RI
4	Aspect	Aspect	Aspect	Aspect	Band 6
5	Band 6	RI	MSAVI	RI	MSAVI

**Fig. 3:**
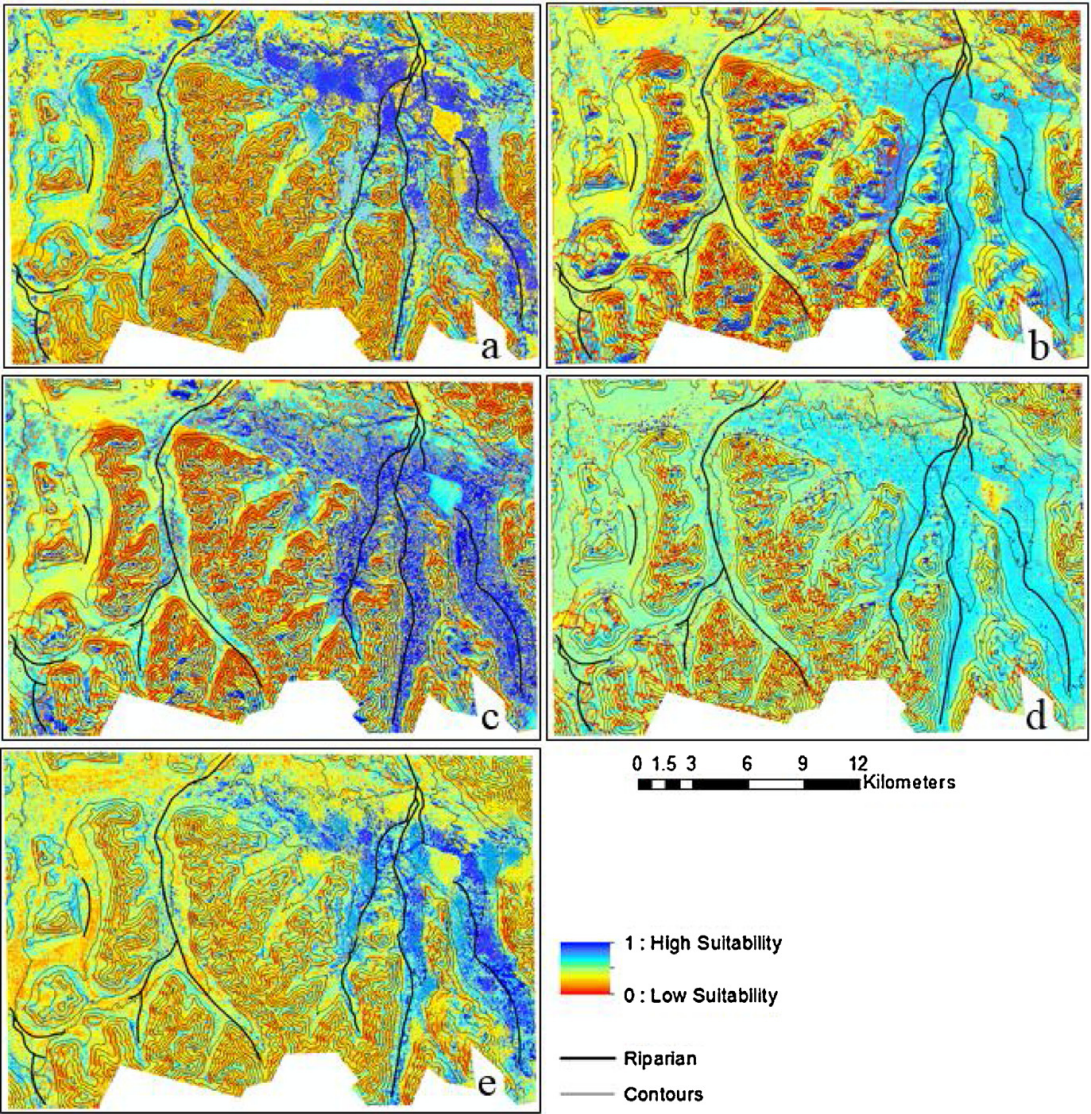
Winter habitat probability maps for the eastern Tajikistan Pamirs derived from five species distribution models: (a) BRT = Boosted Regression Tree, (b) GLM = Generalized Linear Model, (c) MARS = Multivariate Adaptive Regression Splines, (d) Maxent, and (e) RF = Random Forest. Areas near gentler slopes show high probability values (0 to 20°) for winter habitat suitability.

**Fig. 4:**
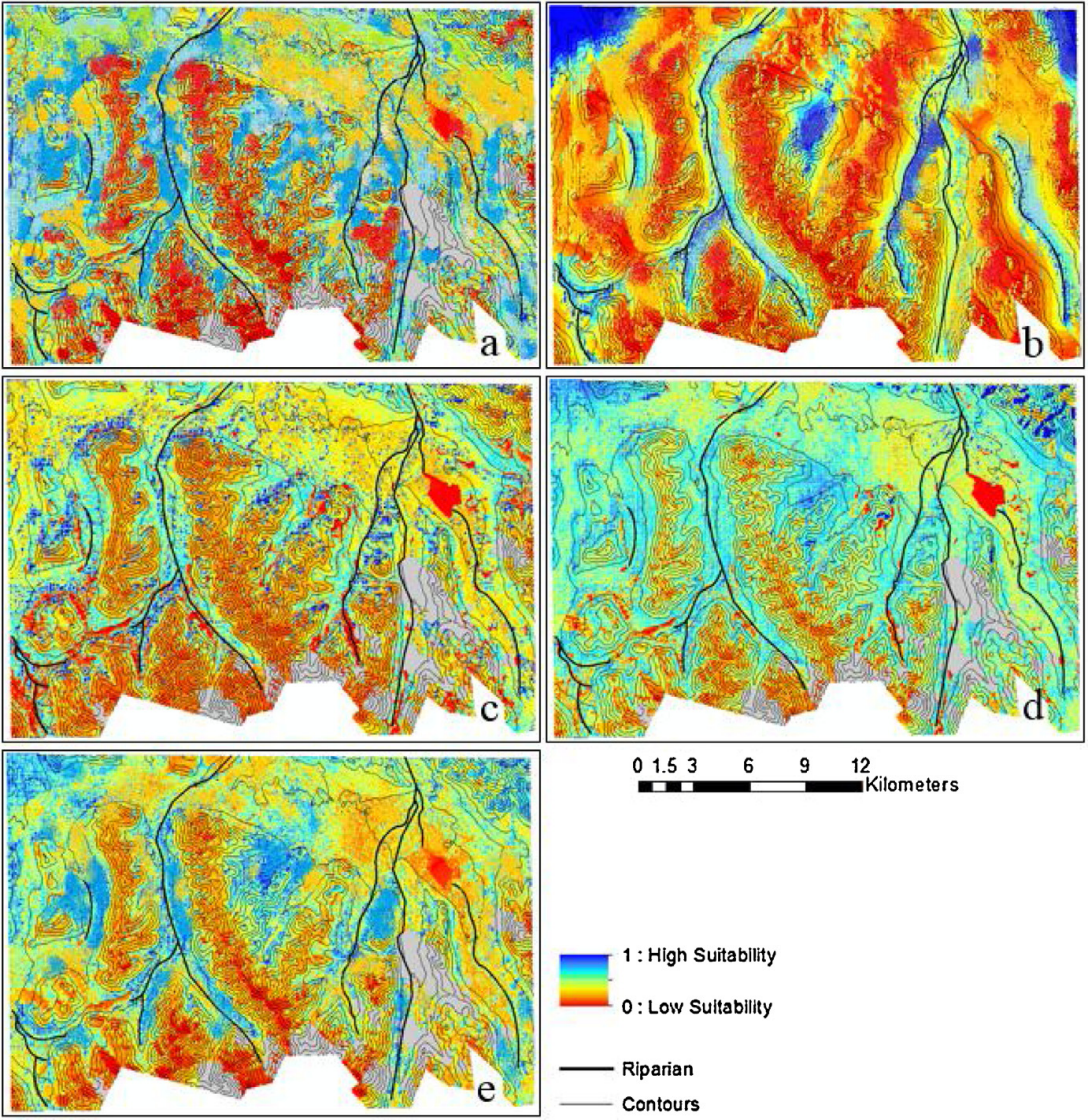
Summer habitat probability maps for the eastern Tajikistan Pamirs derived from five species distribution models: (a) BRT = Boosted Regression Tree, (b) GLM = Generalized Linear Model, (c) MARS = Multivariate Adaptive Regression Splines, (d) Maxent, and (e) RF = Random Forest. Regions near riparian areas show high probability values for summer habitat suitability.

Models for the northeastern part of the study area resulted to high values (dark blue) as shown in the winter habitat suitability maps predicted by the five models (Fig. 3). A close inspection of the maps revealed that these areas with high probability values were on gentler slopes (between 0° to 20°). The summer habitat suitability maps are clearly differentiated from winter habitat maps (Fig. 4). The models produced high probability values for summer habitat near riparian areas. The two most important predictors of summer habitat were the distance to riparian areas (proximity to rivers and streams) and the NDVI (greenness value > 0.4) (Table 3).

**Fig. 5:**
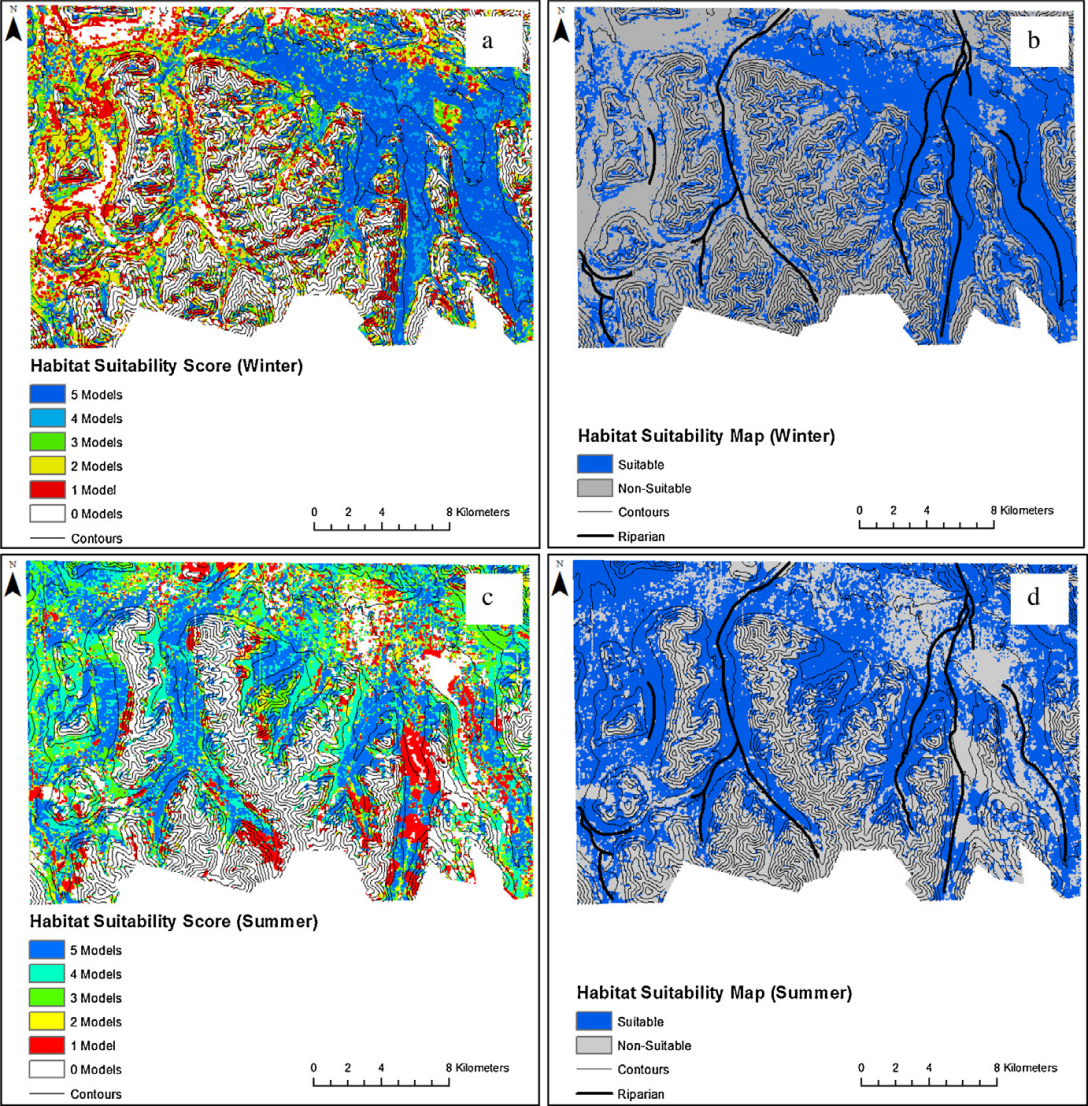
Combined individual habitat suitability maps for argali wild sheep in the southeastern Tajik Pamirs derived from five species distribution models for (a) winter and (c) summer. A high score of 5 indicates all SDMs assigned that pixel contain suitable habitat for the species. Suitability maps based on agreement of at least 3 models are shown in (b) for winter and in (d) for summer. Models are in concordance (shades of blue) that regions surrounding riparian areas are highly suitable for argali for summer, while regions of gentler slopes are highly suitable for argali for winter.

A combination of the five models are shown in Fig. 5a and c. All five models agreed that the habitat suitability for argali on winter was in the northeastern region (Fig. 5a). The sites surrounding riparian areas (Fig. 5c) were habitats highly suitable for argali for the summer season. Sites with at least 60% of the algorithms that were in agreement (colored green and cyan) can be found on the edges of the blue (5-model agreement). Sites with two or less models in agreement were located farther from the suitable areas. Fig. 5b and d depicted the habitat suitability map for argali in the winter and summer, respectively, as predicted by the agreement of at least three SDMs. The combination of three models predicted a suitable habitat of 161.4 km^2^, about 30% of the total study area.

## 4 Discussion

This study is the first seasonal habitat suitability modeling for Marco Polo argali. We observed that the contribution of topographic variables varied in predicting summer and winter suitable habitats for argali. Slope and aspect were better predictors for the winter than summer model, whereas RI was included in the top five predictors for both models. We expected RI to be a significant predictor of habitat because the variable is, in part, defined by DEM. Contrary to our expectations, the model did not select Landsat bands 3 (red) and 4 (NIR) that have more relevance to vegetation and soil features (Salas et al., 2016b). This maybe because the NDVI and MSAVI were both calculated from bands 3 and 4. In fact, all other bands except band 6 (temperature) were not predictive of habitat selection. The association of argali to temperature variable was also reported in Khan et al. (2015). Our results confirmed a study for argali in Tibet (Singh et al., 2009) that also showed NDVI and riparian locations as the strongest predictors for summer habitats.

### 4.1 Winter habitat suitability model

Winter argali distributions are depicted in Fig. 3. During winter, male and female argali were clustered mostly near gentler slopes between 0° to 20° and between elevations of 4200 m to 4300 m (Fig. 5a and b). Although there are elevations as high as 5200 m in the study area (Salas et al., 2015), argali avoided these higher regions in winter and preferred lower elevations and valley floors probably to avoid deep snow and to find forage. Studies by Tilton and Willard (1982) and Jalkotzy (2000) also showed movement of bighorn sheep to much lower areas of the winter range due to less snow accumulation and much warmer temperatures than higher elevations. In our study area, the temperature of the predicted suitable winter habitat for sheep ranged from −17 °C to −21 °C. The higher elevations that were classified nonsuitable winter habitat reached a minimum temperature of −30 °C. Temperatures at high altitudes were probably the major factor influencing the winter habitat suitability for argali in the Pamirs. These indirect and direct effects of temperature on ungulates could explain why the thermal band of Landsat (band 6) was one of the important variables that dictated the winter habitat model. Our inclusion of all the Landsat parameters in the modeling have allowed us to identify the key Landsat band for argali habitat selection, well beyond the topographic variables used by most studies. Areas without argali presence near suitable habitat could be interpreted as underutilized habitat based on the habitat criteria.

Aspect appeared to be a key variable in our winter model, with argali preferring south-facing mountain slopes during winter. These model results agreed with those of Habib (2008) for argali in the Wakhan region of Afghanistan, and Chetri and Pokharel (2005) for argali in Tibet. Their results showed that more than 50% of the observed argali populations preferred south-facing slopes, followed by the west and north-facing slopes. Argali populations in Mongolia also preferred southern slopes with low snow coverage (Mandakh et al., 2005).

### 4.2 Summer habitat suitability model

As per model result (Table 3), the two most significant variables that determine suitable habitat in the summer model were distance to riparian areas (water accessibility) and NDVI (greenness). Salas et al. (2015) showed that 42.28% of the vegetation cover in the study area was within 1 km from the riparian zone. The availability of forage and the distribution of argali were shown to have a strong correlation, as also observed by Khan et al. (2015). Also, the low vegetation height (≤ .5 m) in riparian areas does not provide hiding cover for predators thus reducing predation risk. Habitat alterations that decrease the amount of forage in areas in proximity to riparian areas would likely decrease the amount of suitable summer habitat for sheep. Disturbance factors that could negatively affect access to riparian areas include sport hunting which only occurs in winter, poaching and presence of herder camps in valleys and their dogs. Andrew et al. (1999) tested the hypothesis that North American bighorn sheep distribute themselves near riparian habitat and concluded that sheep sightings occurred nearest to water sources where available forage was abundant.

The suitable summer habitat for argali in the eastern Pamirs confirmed our previous results (Salas et al., 2015). These habitats were within 1 km from riparian areas, at elevation of 4200 m to 4400 m, and close to escape terrain. Escape terrain is often defined by slope or terrain ruggedness and hence may not be a significant habitat predictor as they are often highly correlated (McKinney et al., 2003). In the absence of the RI variable, escape terrain would be a significant predictor of summer habitat. The suitable areas for summer in our study area were primarily gentler slopes (0° to 15°), as also observed by Singh et al. (2009), Chetri and Pokharel (2005), and Namgail et al. (2004) for summer studies of argali habitat in Tibet. The response of the sheep to slope is probably related to the abundance of green vegetation located near riparian areas. Also, Singh et al. (2010) highlighted that gentle slopes in open landscapes could provide higher grounds allowing a higher visibility to scan for predators. A summer habitat study of bighorn sheep at the Sonoran Desert in southeastern California (Gionfriddo and Krausman, 1986) also showed sheep used habitats in or near escape terrain, although in our model distance to escape terrain was not a significant predictor for argali habitat suitability.

### 4.3 SDM performance and caveats

We expected different results for suitable habitat for each season from the five SDMs as previously reported (Capinha and Anastácio, 2011; Robert et al., 2016). No models had calibration plots that were without some form of over or underprediction. Biases were observed when predicted values were higher/lower than the observed probabilities of occurrence. Problems inherent in each algorithm due to differences in model assumptions and algorithms were resolved through the summation of individual predictions. Combining results from various models would yield lower mean error than any of the constituent individual results (Araújo and New, 2007). Here we summarized five binary maps and only presented one habitat suitability map for summer and one for winter, underscoring the agreement among models.

Among the five models, BRT was the best model for winter habitat. It outperformed the other four in measures of model performance. BRT delineated the higher slopes in steep mountain sides from the much gentler slopes at lower elevations. Like the winter model, BRT could be the best SDM for summer habitat. It outperformed the other four in terms of the measures of AUC performance. BRT clearly distinguished riparian areas from areas in unsuitable steep mountain slopes. Our results indicated that BRT’s ranking of predictors could offer information essential in studying the basic ecology of argali that are otherwise hard to attain. Next to BRT, Maxent also showed high AUC numbers for both summer and winter models. In contrast, RF was the underperforming model for winter. No clear distinction between low and high probability of suitable winter and summer habitat was evident, especially in the western section of the study area. GLM and RF were the two underperforming algorithms for summer habitat modeling.

The strength of the results depended on both the selected predictors as well as the methodology in building the SDMs. Here, we chose predictors that were relevant for the argali and ran individual models. This study also excluded temporal bioclimatic data as habitat predictor. While bioclimatic data could add extra explanatory power into the models, we chose to disregard it because of the minimal differences in values (e.g., minimum and maximum precipitation) observed throughout our study area and also because of the lack of available climate data in sufficient resolution. Unless applied to a wide region, the addition of the bioclimatic data would not bias the results. Also, we did not test the transferability of our habitat-based predictive distribution models to new areas. However, since our models were based on variables that are a mix of functional species-specific resources, they are likely to have high transferability. Predictive distribution models based on essential functional resources could transfer better in space (Vanreusel et al., 2007). Since our models did not extend beyond the conditions represented by the data (Wenger and Olden, 2012), transferability assessment is not necessary.

Finally, we used a greenness data that was derived from a medium-course spatial resolution Landsat. In the study area, there could be palatable plants that may have not been detected by Landsat-derived indices (e.g., NDVI, MSAVI). Higher spatial resolution images would have been preferable in mapping greenness.

## 5 Conclusion

Our results suggest that spatially explicit statistical models could help in the understanding of the factors affecting seasonal habitat selection by argali. Further, our results indicate that management of argali habitat should not only focus on the current suitable habitat of Marco Polo sheep as presented in our models, but also the adjacent, unused, and potential summer and winter habitats identified by the models. These habitats might be avoided because of poaching and presence of herders. Active herder camps as a predictor of habitat use in the habitat suitability model would be useful. A future research challenge should be directed at verifying the benefits of these potential and underutilized habitats. Also, movements of sheep from the winter range to the summer range needs study. The elevational movements appear to be an attempt to optimize constraints over an array of different conditions (Festa-Bianchet, 1988). Timing of movements from one habitat to another has been documented with linear distances of migration varying in relation to the distribution of suitable habitat (Shackleton et al., 1999). Studying argali sheep movements at higher elevations of the Pamirs poses a challenge to future studies.

## Declarations

### Author contribution statement

Eric Ariel L. Salas: Conceived and designed the experiments; Performed the experiments; Analyzed and interpreted the data; Contributed reagents, materials, analysis tools or data; Wrote the paper.

Raul Valdez, Stefan Michel: Contributed reagents, materials, analysis tools or data.

### Funding statement

This work was funded by Safari Club International Foundation (SCIF) and the Federal Government of Germany via Deutsche Gesellschaft für Internationale Zusammenarbeit GmbH.

### Competing interest statement

The authors declare no conflict of interest.

### Additional information

No additional information is available for this paper.
